# Perspectives on COVID-19 vaccination for pregnant women in South Africa

**DOI:** 10.4102/phcfm.v13i1.2998

**Published:** 2021-07-27

**Authors:** Mehreen Hunter, Jagidesa Moodley, Neil Moran

**Affiliations:** 1 Western Cape Department of Health, Cape Town, South Africa; 2 School of Public Health and Family Medicine, Division of Public Health Medicine, University of Cape Town, Cape Town, South Africa; 3 Women’s Health and HIV Research Group, Durban, South Africa; 4 Department of Obstetrics and Gynaecology, School of Clinical Medicine, Faculty of Health Sciences, University of KwaZulu-Natal, Durban, South Africa; 5 KwaZulu-Natal Department of Health, Pietermaritzburg, South Africa

**Keywords:** COVID-19, vaccination, pregnancy, arguments, women

## Abstract

Coronavirus disease 2019 (COVID-19) is a pandemic that has created a global health crisis and upended conventional methodologies, both in the governance and clinical structures of Health Care Systems. The spread of COVID-19 has necessitated a coordinated public health response in an effective, extensive and expedited vaccination rollout strategy with the ultimate aim of limiting all nidi of infection for the pathogen. For this goal to be realised, pregnant women, as a cohort, cannot reasonably be excluded from this initiative, despite the initial reluctance to include them in clinical trials for various ethical and legal reasons. Weighing the detrimental complications of COVID-19 on maternal and perinatal outcomes against the hypothetical risk of vaccination in the context of promising, albeit indirect, safety and efficacy data, this report argues that all pregnant women should be offered the choice of whether or not to receive the COVID-19 vaccine based on the available evidence and their individualised risk-benefit ratio.

## Background

The coronavirus disease 2019 (COVID-19) is a pandemic that has created a global health crisis that necessitated a public health response to limit all nidi of infection. South African data from 2020 demonstrated a 30% increase in maternal deaths since the pandemic started.^1^ Whilst most of these excess deaths are likely from the indirect effects of COVID-19 on maternal care,^1^ various women’s health organisations such as the ‘International Federation of Gynecology and Obstetrics (FIGO)’ and the ‘Centers for Disease Control and Prevention (CDC), have deemed pregnancy a risk factor for severe COVID-19.^2,3^ This report argues that all pregnant women should be offered the choice of whether or not to receive the COVID-19 vaccine based on the available evidence and their individualised risk-benefit ratio.

## Effect of COVID-19 on pregnancy

Because of the immunosuppressive and cardiorespiratory changes of pregnancy, pregnant women are postulated to be particularly susceptible to COVID-19. Heightened maternal metabolism, gestational anaemia and fetal oxygen consumption can lead to physiological dyspnoea which can be difficult to distinguish from COVID-19 infection, resulting in delayed diagnosis and uninhibited community transmission.^4^ Whilst the majority of COVID-19 infections in pregnancy are asymptomatic^5^ and the absolute risk for extreme negative outcomes such as ICU admission, mechanical ventilation and death is low, observational data suggests that pregnant women are at risk of severe illness, particularly in their third trimester.^5,6,7^ Despite this, pregnant women were not part of initial COVID-19 vaccine trials. Fortunately, initiatives to involve pregnant women in COVID-19 vaccine trials are underway.^3^ As of February 2021, the first vaccine trial including pregnant women had commenced.^8^

Severe acute respiratory syndrome coronavirus 2 (SARS-CoV-2) infection in pregnancy has been linked to an increased incidence of preeclampsia, preterm birth (mostly iatrogenic for maternal and fetal compromise),^4,5^ stillbirth, thromboembolic disease and caesarean delivery.^6,9^ Vertical transmission remains a topic of debate with current available data suggesting an approximate 2% – 3% risk of transmission.^9^ However, the World Health Organization cautions that, as a result of a lack of standardised definitions and adequate diagnostic tests, further study needs to occur.^10^

**TABLE 1:** Standpoints of various international bodies regarding the administration of COVID-19 vaccines to the pregnant cohort of the population.

International body	Standpoint on vaccination in pregnancy
American College of Obstetricians and Gynaecologists	COVID-19 vaccinations should not be withheld from the pregnant population.^11^
Royal College of Obstetricians and Gynaecologists	COVID-19 vaccines should be considered for pregnant women at high risk of viral exposure or complications because of comorbidities, but should also be offered to all pregnant women in tandem with the rest of the population, in line with vaccine roll-out strategies, without discrimination.^12^
International Federation of Gynaecology and Obstetrics	COVID-19 vaccination should be offered to pregnant and breastfeeding women as there are no actual or theoretical risks from the vaccines that outweigh the potential benefits.^2^ Following a risk-based approach and restricting access to the vaccine to those with comorbidities or at high-risk of exposure may disadvantage the rest of the pregnant cohort, who are, by definition, at risk of severe COVID-19 infection.^2^
The United States Centers for Disease Control and Prevention	Any currently authorised COVID-19 vaccine (Pfizer-BioNTech, Moderna or Janssen) in the United States, without preference, can be offered to pregnant individuals.^3,14^
The World Health Organization	The COVID-19 vaccine (Janssen, Moderna or Pfizer-BioNTech) should be offered to pregnant women, with informed consent, if the benefit of the vaccine outweighs the risk, that is in healthcare workers or those with comorbidities. Delaying pregnancy because of vaccination or testing for pregnancy prior to vaccine administration is not recommended.^16,17,18^
South African Society of Obstetricians and Gynaecologists	COVID-19 vaccines can be offered to pregnant women who are at high-risk of disease acquisition or the development of severe illness. Vaccination should not be avoided in women contemplating pregnancy.^13^

COVID-19, coronavirus disease 2019.

## Evidence of vaccine safety

Data on the safety of the COVID-19 vaccinations in pregnancy is limited. However, animal tests done by BioNTech, Moderna and Janssen revealed no safety concerns surrounding female fertility, fetal, embryonal or postnatal development.^2,3,11,12,13,14^ The vaccination technologies employed by Janssen have been used previously for other vaccine programmes, such as the Ebola vaccine trial, that included a pregnant cohort, irrespective of trimester. No adverse pregnancy or infant outcomes related to the vaccine were found.^3,11^

Administration of the COVID-19 vaccine in pregnancy can result in side-effect profiles similar to those experienced by non-pregnant individuals.^3,8^ There is no evidence of male or female infertility resulting from receiving the vaccine nor is there evidence of fetal risk when pregnant women are vaccinated with non-replicating vaccines.^3^ As the current vaccines on offer are non-replicating and cannot cause infection, experts believe that they are unlikely to pose a risk either to the mother or her fetus.^3^ Nevertheless, some uncertainty still remains in the absence of solid safety data only afforded by a clinical trial. Women who have fallen pregnant during or just prior to clinical trials are being followed up by vaccine manufacturers.^3^ More than 20 000 pregnant women received the COVID-19 vaccine in the United States and there were no ‘red flags’ from those patients thereafter.^15^ A small study assessing COVID-19 messenger RNA (mRNA) vaccination in pregnant and lactating women revealed that the vaccines induced immune responses stronger than that of natural infections and immunity was transferred to neonates transplacentally and via breastmilk.^8^

## Recommendation

Whilst data is reassuring, it is imperative that pregnant women and those planning a pregnancy are counselled appropriately and allowed to make an informed decision as to whether or not they choose to get vaccinated. The ethical points of maternal autonomy and distributive justice must be considered. Advice from a trusted healthcare provider is an important factor in the decision-making process for mothers.^19^ It should be explained that:

- Whilst the majority of individuals who develop COVID-19 in pregnancy are asymptomatic,^5^ pregnancy is a risk factor for severe COVID-19 illness.^3^
- The expected side effects and rare complications need to be discussed and it should be noted that they are not dissimilar from the non-pregnant cohort.^3,8^
- There is no evidence that the current COVID-19 vaccines in use in South Africa (Pfizer or Janssen) pose a risk either to the mother or her fetus and there is no need to perform a pregnancy test prior to administration of the vaccine nor does one need to delay pregnancy as a result of having taken the vaccine.^3^
- There is evidence of a robust immune response following mRNA vaccination as well as immunity transfer via breastmilk.^8^
- For those wanting to conceive, there is no evidence that the COVID-19 vaccines, currently in use, cause infertility.^3^

However, data is currently limited and, whilst promising, more studies need to occur. Therefore, every decision should, ideally, be taken in consultation with a healthcare provider so that the risks and benefits can be adequately considered. Whilst highly recommended, this step need not be mandatory as it should not become a barrier to receiving the vaccine.^11^ Pregnant women should be counselled regarding the best available evidence, international recommendations, safety profiles, the risks and benefits and that there is some information which is still currently unknown.^11^

It is our recommendation that South Africa, in line with the International Federation of Gynaecology and Obstetrics, the American College of Obstetricians and Gynecologists and the Centers of Disease Control and Prevention, take the decision to allow all pregnant women, and those of childbearing age, the choice to receive a COVID-19 vaccination regardless of comorbid status or trimester on the background of informed consent. This decision should, ideally, be taken in conjunction with the maternal healthcare provider who should provide adequate counselling and assist her in assessing and personalising the risk-benefit ratio to her specific circumstance.^20^ Maternal autonomy is paramount and her decision, either in accepting or declining a COVID-19 vaccine, should be supported and respected.^3,11^
